# Artificial intelligence bias in the prediction and detection of cardiovascular disease

**DOI:** 10.1038/s44325-024-00031-9

**Published:** 2024-11-21

**Authors:** Ariana Mihan, Ambarish Pandey, Harriette G. C. Van Spall

**Affiliations:** 1 Department of Medicine, Faculty of Health Sciences, McMaster University, Hamilton, Canada; 2 University of Texas Southwestern, Dallas, TX USA; 3 Population Health Research Institute, Hamilton, ON Canada; 4 Baim Institute for Clinical Research, Boston, MA USA

**Keywords:** Cardiology, Health care

## Abstract

AI algorithms can identify those at risk of cardiovascular disease (CVD), allowing for early intervention to change the trajectory of disease. However, AI bias can arise from any step in the development, validation, and evaluation of algorithms. Biased algorithms can perform poorly in historically marginalized groups, amplifying healthcare inequities on the basis of age, sex or gender, race or ethnicity, and socioeconomic status. In this perspective, we discuss the sources and consequences of AI bias in CVD prediction or detection. We present an AI health equity framework and review bias mitigation strategies that can be adopted during the AI lifecycle.

## Background

Digital healthcare data are rich resources that can be analyzed to understand and, importantly, enhance cardiovascular care^1^. Diagnostic test results, electronic health records, and digital technologies such as mobile devices, apps, and wearable or intracardiac devices can serve as data repositories to train artificial intelligence (AI) algorithms to further improve care. AI has the potential to identify risk factors, predict disease, detect early stages of disease, and potentially identify patients who could benefit from early interventions that could change disease trajectory^2–6^.

Inequities related to the digital divide and healthcare services disproportionately affect historically marginalized populations, such that some groups are inadequately or inaccurately represented in digital datasets or receive substandard care^7–11^. AI bias can arise when such data are used to train algorithms or at any step in the process, from development of the study question to data handling to model development, testing, implementation, and algorithm development and post-deployment clinical practices^12–14^. These biases can be amplified in subsequent cycles of AI learning and cause clinical harm, further exacerbating inequities amongst those already facing them^15^. In this perspective, we discuss the sources and consequences of AI bias in CVD prediction or dection, and present strategies to mitigate AI bias and optimize model performance in all people.

## Literature search

We used a broad search strategy to identify studies related to the applications of AI in CVD prediction and detection on PubMed and Google.^16^ Our literature search, conducted on OVID Medline and Embase, combined terms related to artificial intelligence (e.g., “artificial intelligence” OR “deep learning” OR “algorithm*”), bias (e.g., “bias” OR “disparities”), cardiovascular disease (e.g., “cardiovascular disease*” OR “heart disease” OR “heart failure” OR “vascular disease*”) and prediction (e.g., “prevention” OR “screening” OR “prediction” OR “risk assessment*”). In addition to keywords, we also included relevant corresponding MeSH terms in the search strategy. We identified six studies with examples of AI bias in CVD prediction and detection. We identified bias mitigation strategies through searches of the grey literature. We synthesized information from all relevant articles narratively.

## The role of AI in assessing risk, predicting, and detecting CVD

Across the world, there is a disproportionate burden of CVD and cardiometabolic risk factors in resource-poor regions, socioeconomically deprived groups, and some ethnic minorities. These groups also face healthcare disparities^17^. CVDs result from cumulative exposures and risk factors throughout the lifetime, which could be targeted with screening and early intervention^18^. AI may enhance preventative efforts through risk assessment, disease prediction, and early detection.

**Fig. 1:**
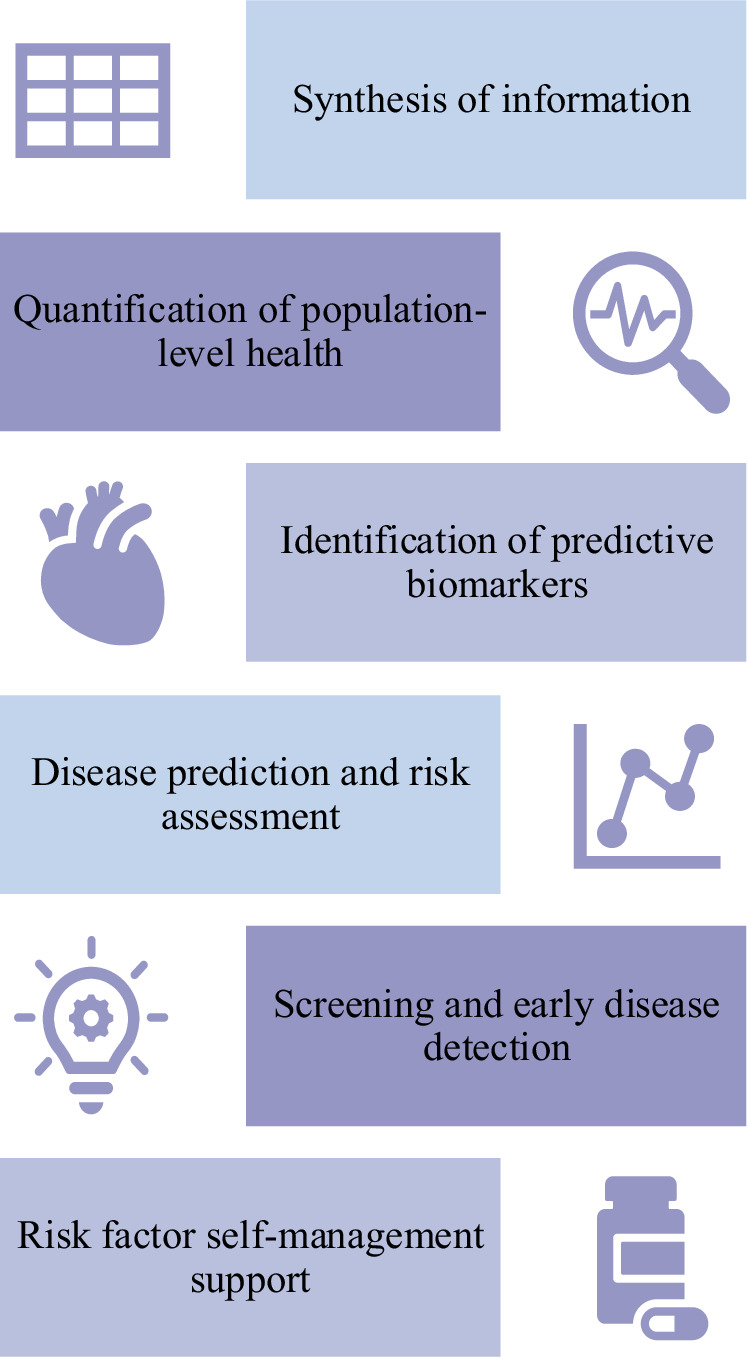
Applications of AI in predicting and detecting CVD.

AI can facilitate the prediction, identification, and management of CVD and its risk factors, including obesity, hyperglycemia, dyslipidemia, and hypertension (Fig. 1)^3,19^. For example, in a cohort study that analyzed 1066 participants, a mobile application that integrated wearable device data, machine learning (ML), and continuous glucose monitoring was associated with improvements in participants’ metabolic health (such as glycemic levels, variability, and events)^4^. Further, AI algorithms may perform more favorably than traditional risk scores; in a multi-center cohort study, an exposome-based ML model outperformed the Framingham risk score for CVD risk estimation (AUC = 0.77 versus AUC = 0.65)^5^. Other studies have demonstrated the superior performance of ML-based models in predicting the risk of incident heart failure (HF) when compared to existing traditional HF risk scores^20,21^. For example, in a cohort study of 8938 patients with dysglycaemia, an ML-based risk score exhibited superior discrimination and calibration in predicting the 5-year risk of HF when compared to existing HF risk scores (i.e., PCP-HF, MESA-HF, and ARIC-HF)^21^. AI can calculate disease risk and estimate how lifestyle modification (such as increasing physical activity or decreasing BMI) may affect such risk^22^. AI-enhanced ECG models can also be trained to identify biomarkers associated with a greater risk of cardiometabolic disease^23^. On a population-level, AI can quantify the cardiometabolic health of the general population^6^, and identify groups at higher risk of CVD^24,25^. Overall, AI may facilitate reliable prediction and targeted interventions for those at greatest risk of CVDs. AI could also improve health equity in disease screening and detection. For example, in a retrospective study of over 17,000 patients with diabetes, primary care sites that deployed autonomous AI for diabetic retinopathy testing (versus sites that did not deploy AI) experienced increased annual testing adherence in Black patients^26^. AI-deployed sites also experienced better adherence in socioeconomically deprived sub-populations^26^.

## Risks of a digital data divide

The same socioeconomic conditions that influence the risk of CVD may also impact access to digital health technologies^1^. Broadband penetration and access to digital technologies vary across regions^27^, with disproportionate disparities in rural and remote regions and neighborhoods with predominantly ethnic minorities^28^. Access and affordability may be further limited by cognition, language, or digital literacy levels ^27^, which vary across age, ethnicity, or socioeconomic status. In some countries, cultural norms and laws result in gender-specific disparities limiting women’s internet access or digital technologies^27^. Gaps in access to digital technology deprive patients of the direct benefits of digital healthcare and limit their representation in digital datasets. Women, socioeconomically deprived people, ethnic minorities, and those with intersectional identities may also be misrepresented in digital datasets due to biases in the way they are described or the healthcare services that they receive^7,29^.

## Sources of AI bias

AI algorithms can vary in performance across demographic groups, particularly in groups that are under-represented or misrepresented in training datasets or that are subject to discrimination in the healthcare system. Several sources of bias can culminate in AI algorithm bias, defined as systematic and repeated errors that produce unfair outputs and can compound existing inequities in healthcare systems^30^. These types of bias can arise at any step in the AI algorithm development or implementation process and result in health inequities^31^.

Biases can occur in the selection and management of training data. Sampling bias^14^ can result from a homogenous dataset or a biased or narrow group of variables, while representation bias can arise if the training dataset does not adequately represent the target population^32^. For example, AI trained on acute care data may be biased towards more severe cases^33^. Similarly, if an AI-based screening tool is trained on a sample with optimal access to healthcare, but intended for use in a population with variable access to care, the algorithm performance may be biased^31^. These concepts apply to conventional statistical models too; decision-support algorithms and risk-prediction models can perform unequally across sub-populations, depending on who the models were derived and validated in. For example, an in-silico cohort study found that the pooled-cohort equation for CVD risk estimation resulted in substantially different predicted 10-year CVD risks between Black and White patients with the same risk factor profiles^34^.

Measurement bias can result from training datasets that use inaccurate diagnoses (e.g., due to use of inaccurate reference standards)^31^ or that use device readings or equations that perform sub-optimally in some groups relative to others^13^. Variable selection bias may occur if inappropriate predictor variables are chosen, or if important variables (such as socioeconomic determinants of health) are not included in the training dataset. For example, CVD risk-prediction equations that solely include variables that are biological determinants of CVDs without considering social determinants of health may underpredict CVD risk in socially deprived populations^35^. Annotation bias could arise when data labels are applied by clinicians to different populations in a non-objective or unfair manner (such as during annotation of diagnostic data)^36^. Outcome labeling bias can result when an outcome is not consistently obtained or defined across groups^13^. For example, female and ethnic minority patients are more likely to have missed or delayed diagnosis of CVDs^37^, so algorithms trained on such datasets would perform sub-optimally in detecting CVD in these groups. Biases in healthcare processes—for example, in the referral for specialist care, diagnostic testing, or treatment prescription—based on a patient’s demographic characteristics can be propagated in training datasets that inform AI algorithms, resulting in biased recommendations. Algorithmic bias can also arise from how algorithms are used in practice and how they learn once implemented^12^. Evaluation bias may occur if inappropriate metrics are used to assess algorithm performance^32^. Latent biases describe biases waiting to happen when an AI algorithm that is initially fair is prone to developing biases over time^12^ by learning biased clinical practices, interacting with homogenous clinical populations, or prioritizing one type of outcome over others^14^.

## How AI bias may limit CVD prediction and detection

AI bias can result in unintended consequences that negatively impact care. It can result in missed risk factor identification, delayed or missed diagnoses, or inaccurate risk prediction for certain patient populations. Algorithms can be racist, sexist, or classist. For example, algorithms that predict the risk of CVD might learn from persistent socioeconomic, racial, or ethnic inequities in care, and predict inaccurate outcomes in socioeconomically deprived and ethnic minority groups^12^.

Indeed, AI models can have varying performance across demographic groups, indicative of bias (Table 1). For example, a multi-center cohort study assessed an ECG deep learning (DL) model’s ability to detect aortic stenosis, aortic regurgitation, and mitral regurgitation in a sample of 77,163 patients who had undergone an ECG followed by an echocardiogram within 1 year^38^. The model was less accurate in older than younger adults (e.g., ROC AUC = 0.81 in the 18–60 aged group, versus ROC AUC = 0.73 in the 81+ age group), and had numerically worse performance in Black patients (prevalence detected = 4.4%) than White patients (prevalence detected = 10%).

**Table 1:** Examples of AI bias in predicting or detecting CVD

Author (year) Main study aim	Unit of analysis and sample size	AI model and application	How the outcome was established	Biased outcome
Elias et al. (2022)^38^ To assess the performance of ECG deep learning algorithms designed to detect moderate/ severe valve disease	77, 163 patients aged 18+ who had a 12-lead ECG before an echocardiogram at 1 of 3 New York, USA hospitals	A DL model to detect valvular heart disease (AS, AR, MS)	Valvular disease was diagnosed by the echocardiographer	The model had poorer performance in older than younger adults and in Black patients than White patients.
Hong et al. (2023)^41^ To compare the performance of stroke-specific algorithms with pooled-cohort equations developed for the prediction of new-onset stroke across different subgroups and to determine the added value of novel ML techniques	Data from 62,482 adults ≥45 years with CVD risk factors but no history of stroke in four USA cohorts: Framingham Offspring, Atherosclerosis Risk in Communities Multi-Ethnic Study for Atherosclerosis, and Reasons for Geographical and Racial Differences in Stroke	ML stroke-specific algorithms versus traditional stroke prediction models and PCEs	The occurrence of ischemic or hemorrhagic stroke was obtained from the harmonized cohort dataset	All algorithms showed poorer risk discrimination in Black patients than White patients.
Kaur et al. (2023)^39^ To investigate the presence of algorithmic biases relating to age, race, ethnicity, and sex in a DL model trained to predict HF from ECG data and investigate how modifications to the model training and application affect its performance	326,518 ECGs from patients referred for standard clinical indications to the Stanford University Medical Center	A DL model to predict incident HF within 5 years of ECG collection	The incidence of HF was obtained from the EHR.	Model performance was significantly worse in older versus younger patients, and slightly worse in male versus female patients. Among younger patients, the model performance was worse in Black patients compared to other patients.
Li et al. (2023)^40^ To investigate whether ML-based predictive models for CVD risk assessment perform equally across demographic groups and if bias mitigation methods can reduce any model bias	Vanderbilt University Medical Center de-identified EHR: 109,490 adult outpatients with 10-year follow-ups and no previous CVD history	ML-based models to predict the 10-year risk of CVD (coronary heart disease, MI, stroke)	The CVD diagnosis was obtained from the EHR	The ML model demonstrated lower true positive rates and positive prediction values for CVD in female versus male patients. The DL model exhibited sex and race-related biases.

*AI* artificial intelligence, *AR* atrial regurgitation, *AS* aortic stenosis, *CVD* cardiovascular disease, *DL* deep learning, *ECG* electrocardiogram, *EHR* electronic health record, *EMR* electronic medical record, *HF* heart failure, *MI* myocardial infarction, *ML* machine learning, *MR* mitral regurgitation.

Similarly, the performance of a DL model developed to predict incident HF within 5 years of ECGs was tested in a sample of 326,518 patient ECGs^39^. The model performed worse in older (ROC AUC = 0.66) than younger patients (AUC = 0.80). Among younger patients, the model performed worse in Black patients than patients of other racial groups and was particularly worse for Black female patients.

An EHR-based cohort study of 109,490 patients assessed bias via equal opportunity difference (EOD) and disparate impact (DI)) in ML-based predictive models for the 10-year risk of coronary heart disease, myocardial infarction, and stroke^40^. ML models showed bias against female patients; they resulted in lower true positive rates and positive predictive ratios in female patients than male patients, and their corresponding EODs and DIs were significantly higher than reference fairness values (EOD = 0, DI = 1), indicating that the model was more likely to underestimate risk in female patients^40^. The study also examined a DL model that showed significant bias across race (EOD = 0.111, DI = 1.305) and sex (EOD = 0.123, DI = 1.502). De-biasing strategies, such as removing protected attributes, or resampling by sample size, did not significantly mitigate bias across ML models. Resampling by case proportion decreased bias across gender groups, but not race, and decreased model accuracy^40^.

Finally, a retrospective cohort study of 62,482 patients compared the performance of the ML stroke-specific algorithm, existing stroke prediction models, and the atherosclerotic CVD pooled cohort equation^41^. All models exhibited poorer risk discrimination (measured via concordance index (C-index)) in Black patients than White patients^41^. For example, in the CoxNet ML model, C-indexes were 0.70 for Black female patients versus 0.75 for White female patients and 0.66 for Black male patients versus 0.69 for White male patients.

## Mitigating AI bias and promoting health equity in predicting and detecting CVD

**Fig. 2:**
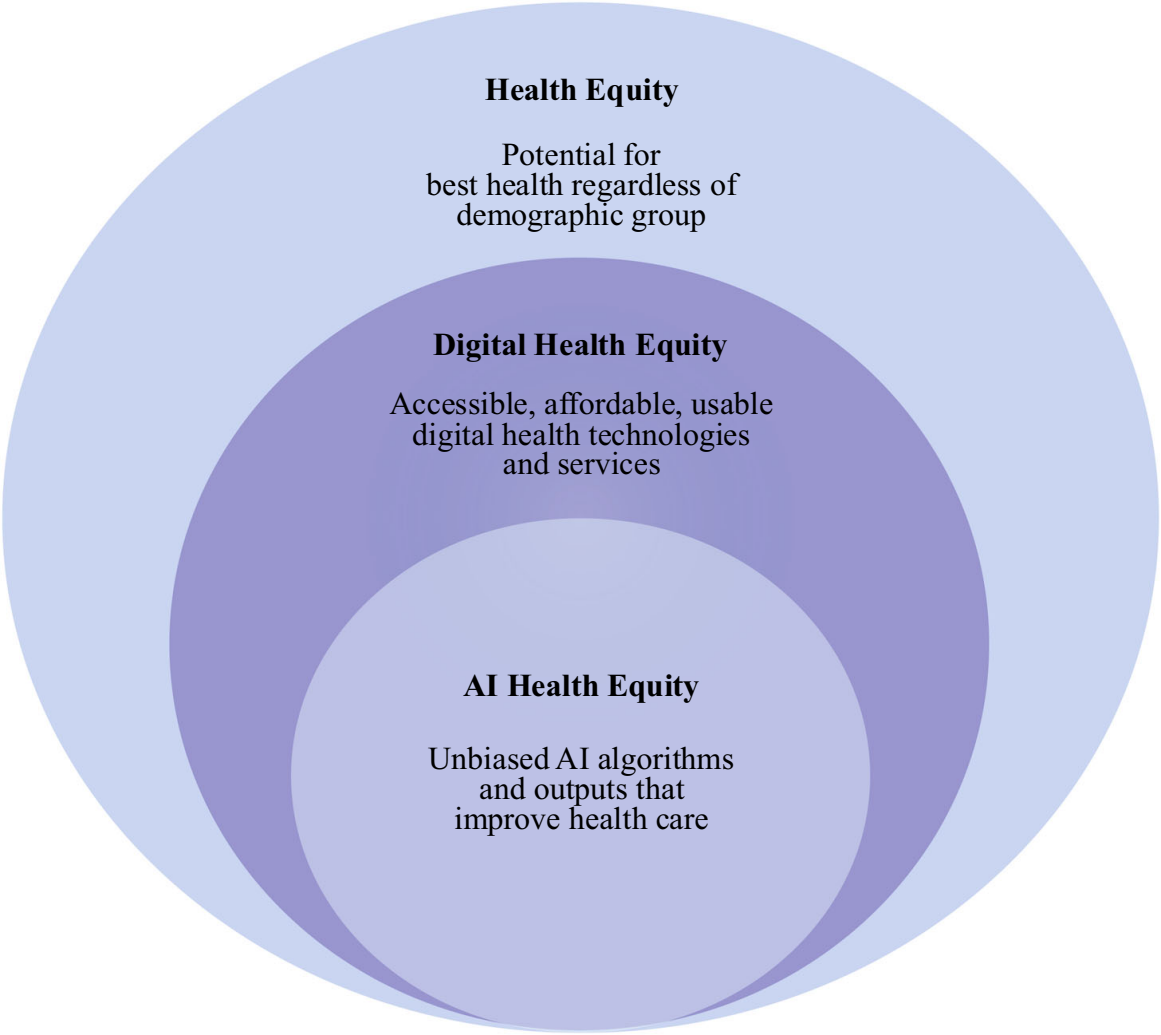
A conceptual framework for AI health equity.

AI algorithms must be developed, trained, tested, and implemented using a health equity lens to meet its potential in CVD prediction and detection (Fig. 2)^16^. An AI equity framework can potentially mitigate disparities and biases that have contributed to inequities across populations.

**Fig. 3:**
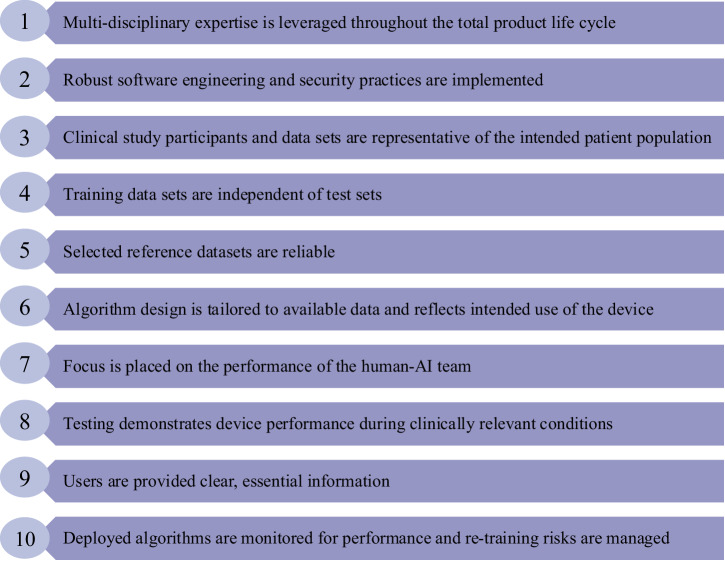
Guiding principles for good ML practice for medical device development: jointly developed by the FDA, Health Canada, and the United Kingdom’s Medicines and Healthcare Products Regulatory Agency^47^.

Guidelines from international, national, and regional regulatory organizations can support efforts in mitigating AI bias. The World Health Organization (WHO) recently outlined priority areas for responsible AI and established the Global Initiative on Artificial Intelligence for Health with United Nations agencies^42^. The WHO has also released several guidance documents surrounding the ethical and governance considerations of AI models. The 2024 guidance document on large multi-modal models (LMMs) identifies potential benefits and risks of LMMs, as well as actionable items for governments to mitigate risks^43^. Some proposed actions include audits and impact assessments following LMM deployment, training to healthcare providers on using LMMs while avoiding bias, and funding for open-source LMMs^43^. Regulatory agencies have also released national guidelines. Examples of this include the US Food and Drug Administration (FDA)’s action plan for mitigating AI bias in medical devices^44,45^ and Health Canada’s draft pre-market guidance for ML-enabled medical devices^46^. Such guidance documents address the lifecycle of ML-enabled medical devices—including components such as design, development, training, testing, and post-market performance monitoring. The FDA, Health Canada, and the United Kingdom’s Medicines and Healthcare Products Regulatory Agency collaboratively identified ten guiding principles for good ML practice for medical device development (Fig. 3)^47^.

**Fig. 4:**
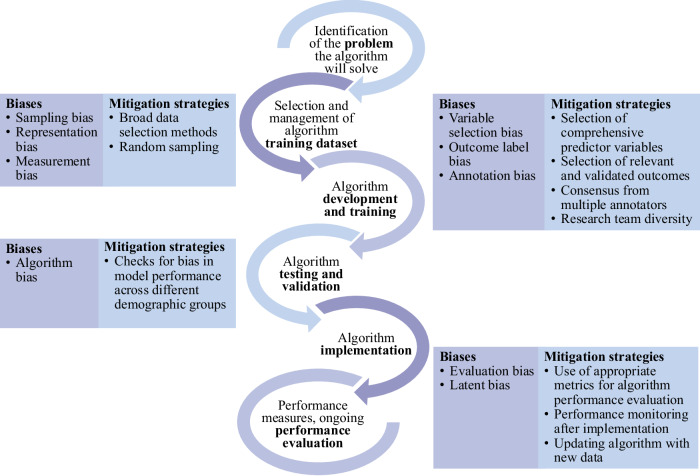
Sources of AI bias and mitigation strategies across stages of algorithm development and deployment.

In addition to following guidelines set out by regulatory organizations, AI researchers and developers could adopt mitigation strategies that address sources of bias arising from stages of the AI algorithm development, training, and testing (Fig. 4)^13,14,16^. Strong efforts should be made to diversify the research team and to engage patients early in the design and development process^13,29,30,43^. Broad data selection methods, including using publicly available datasets^13^ and random sampling, can facilitate representative training data. Selecting or employing strategies to create balanced datasets can help reduce bias. For example, a retrospective study examined AI-algorithms trained on single-lead and 12-lead ECGs to detect paroxysmal/subclinical atrial fibrillation (AF) in patients presenting with sinus rhythm (SR)^48^. Models were trained and tested on two different datasets: a matched dataset (ECGs of patients with AF and an age and sex-matched control group) and a replication dataset (no age and sex-matching)^48^. Because positive cases were overrepresented in older patients in the training data, model performance was unstable across different test-sets; unlike the ECG model trained on the replication dataset, the model trained on the age and sex-matched dataset showed performance consistency across test-sets and when risk factors were included in the model (Table 2). This study demonstrates the importance of ensuring balanced datasets to reduce AI bias.

**Table 2:** Examples of mitigation strategies that reduced AI bias in CVD prediction or detection

Author (year) Study aim	Unit of analysis and sample size	AI application	How the outcome was established	Bias mitigation strategies and outcome
Dupulthys et al. (2024)^48^ To determine if an AI-enhanced ECG with EHR-extracted risk factors can be used to identify subclinical AF during SR in a screening scenario	173,537 ECGs (from 68,880 patients who may have had AF risk factors) from Roeselare, Belgium SR ECGs from patients with and without AF and with or without AF risk factors were analyzed	AI algorithm trained on ECGs with or without confirmed AF and with or without the inclusion of AF risk factors (e.g., previous CVD, obesity, smoking) to detect AF in patients presenting with SR	The diagnostic label of AF or SR was automatically assigned by the *GE MUSE Cardiology Information System*	Dataset balancing (age and sex-matched data). Dataset balancing showed performance consistency across test-sets and when risk factors were included in the model. In contrast, the model trained without age or sex-matching resulted in age bias and unstable model performance across test-sets.
Meng et al. (2022)^50^ To introduce a novel clinical knowledge-enhanced ML pipeline to support timely and cost-effective IHD prediction	Cleveland Clinic Foundation IHD dataset of 303 patients with and without IHD that may have cardiac risk factors	ML-based models to diagnose IHD	IHD was defined as ≥50% narrowing of at least 1 of the coronary arteries on coronary angiography	Clinical input during model development and variable selection. The model with clinical input resulted in superior accuracy compared to the ML models without clinical input.

*AF* atrial fibrillation, *AI* artificial intelligence, *CVD* cardiovascular disease, *DL* deep learning, *ECG* electrocardiogram, *EHR* electronic health record, *IHD* ischemic heart disease, *ML* machine learning, *SR* sinus rhythm.

Comprehensive and relevant predictor variables, including the social determinants of health, could enhance the performance of AI algorithms. For example, in a cohort study of an algorithm that predicted in-hospital mortality, incorporating socioeconomic parameters in the model substantially improved model performance (discrimination, prognostic utility, and risk reclassification) in Black patients when compared with in-hospital HF mortality prediction models that included race without socioeconomic status as a covariate^49^.

Bias can be mitigated by including input from clinicians in model development. An ML-based model enhanced with clinical input better detected CAD than ML models without clinical input^50^. Clinician input during model development identified variables in the dataset that decreased risk discrimination. Removal of these variables resulted in superior accuracy (accuracy = 94.4%) compared to the ML models without clinical input (accuracy ranging from 82.18 to 90.78%)^50^.

Bias from annotation and labeling of outcomes can be mitigated by consensus from multiple annotators during data annotation^36^ and selection of relevant and validated outcomes. When possible, consideration should be given to all-cause outcomes, which do not require adjudication and are less prone to error than disease-specific outcomes. Following this, valid strategies should be used for data cleaning and completeness^13^. Algorithms should be externally validated across different patient demographic groups^13^. Performance should be reported across different population groups in a transparent, timely manner^14^.

Algorithm access, use, and performance should be monitored after implementation, and the algorithm should be updated with new data if needed^14,31^. When AI applications are used in care, clinicians should be transparent with their patients. A qualitative study showed that while many patients were open to AI use in their care, they preferred it as an aid to their provider’s judgment and not the sole basis for decision-making^51^. The WHO has acknowledged that when implemented responsibly, AI has the potential to advance sustainable development goals^42^ as well as healthcare research^52^. However, the risk of bias, transparency, and patient privacy should be carefully assessed and mitigated throughout these processes^52^.

## Conclusion

AI has the potential to improve CVD outcomes by identifying those at high risk of CVD, detecting early disease, and offering timely treatment. However, this potential can be limited by AI algorithmic bias which can disproportionately impact marginalized populations. Bias can stem from several sources, including from the study question, initial training dataset, development and testing of the algorithm, and implementation in practice. These biases could exacerbate existing healthcare inequities and miss opportunities for predicting and detecting CVDs. An AI health equity framework, along with continuous bias surveillance and mitigation, could promote an equitable approach to utilizing AI for CVD prediction and detection.

## Data Availability

No datasets were generated or analysed during the current study.
